# An unusual case of ectomesenchymal chondromyxoid tumour of the left tonsillar bed: imaging and histopathologic features

**DOI:** 10.1259/bjrcr.20150183

**Published:** 2016-07-28

**Authors:** Alessandro Stecco, Martina Quagliozzi, Massimiliano Pino, Paolo Spina, Franco Pia, Renzo Boldorini, Alessandro Carriero

**Affiliations:** ^1^Radiology Department, AOU Maggiore della Carità, Eastern Piedmont University, Novara, Italy; ^2^ENT Department, AOU Maggiore della Carità, Eastern Piedmont University, Novara, Italy; ^3^Pathology Department, AOU Maggiore della Carità, Eastern Piedmont University, Novara, Italy

## Abstract

We describe a case of a rare soft-palatal and parapharyngeal mass in an adult woman. A 71-year-old female presented with a huge mass protruding from the soft palate, complaining about difficulty in swallowing for the past 4 months. After inspection and ear nose and throat fibroscopy, in which the mass appeared regular-shaped and with a regular mucosa, the patient underwent a CT scan and MRI examination. The CT scan showed an oval, not-infiltrating, hypodense mass including a discrete irregular-shaped central calcification, while the MRI examination confirmed the non-infiltrating growth and showed that around the calcified core, the lesion was surrounded by fat. After surgical removal, the histopathologic diagnosis was that of an "ectomesenchymal chondromyxoid tumour". Ectomesenchymal chondromyxoid tumour is a rare benign neoplasm arising from the tongue. Although only 45 cases have been reported in the literature, there are several unique features that define this lesion. Ours is the first case with a complete CT scan and MRI with diffusion imaging description.

## Background

This case has specificity for both imaging description and anatomical localization: the tonsils are an extremely rare site for an uncommon type of tumour; in fact, the majority of the 45 cases of ectomesenchymal chondromyxoid tumours (ECMTs) described in the literature were localizeda th lingual site.

## Case presentation

A 71-year-old female presented to an ear, nose and throat specialist in November 2014 complaining of obstructive dysphagia for 4 months, without pain. She did not report haemoptysis or other clinical signs or symptoms.

During the clinical examination, the physician found a bulky mass localized to the left tonsillar bed, without the presence of mucosal ulceration. There was no clinical evidence of any pathological cervical lymph nodes. A maxillofacial CT scan and MRI were then scheduled.

## Investigations

**Figure 1.**
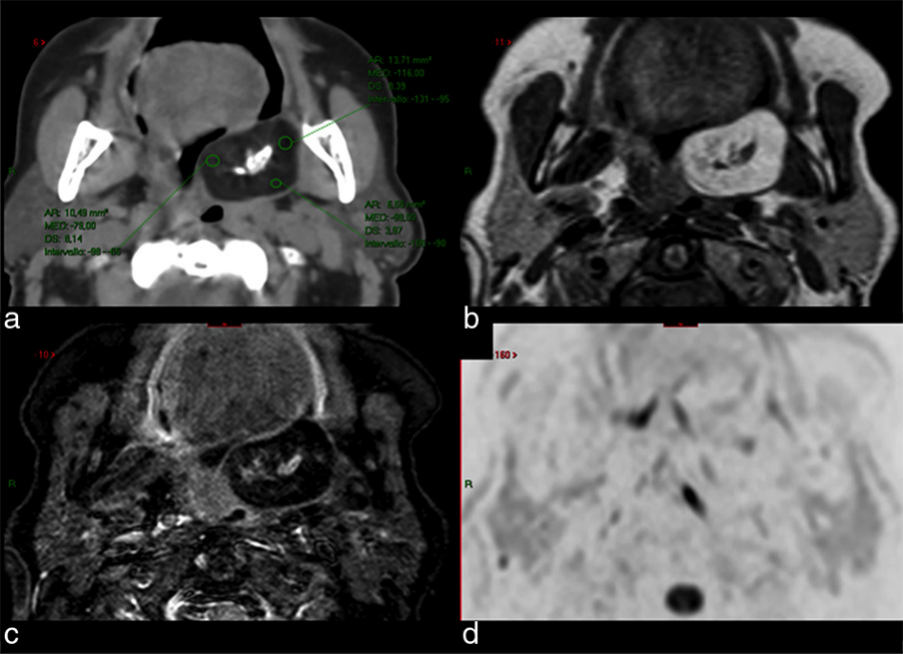
CT scan (a) and MRI [(b) axial *T*_1_ weighted; (c) axial STIR; (d) axial diffusion-weighted imaging with background suppression) show a juxtatonsillar left parapharyngeal mass with central calcification and fat tissue peripherally. Hounsfield units in the CT scan ranged between –80 and –116 (a), while fat saturation by means of STIR technique (c) confirmed the presence of fatty tissue. CT scan after ontrast media showed no enhancement (not shown). STIR, short tau inversion-recovery.

The patient underwent a CT scan with and without contrast media (multislice, 8 rows), and a concomitant MRI (1.5 T, with a head and neck coil), which showed a huge oropharyngeal mass with a maximum axial diameter of 3.5 cm, in the left tonsillar bed and prestyloid parapharyngeal space, with heterogeneous tissue structure owing to the presence of fatty and calcified areas (Figure 1); no areas with contrast enhancement were detected.

This lesion caused dislocation of the soft palate with associated subtle contralateral compression on the ipsilateral pterygoid muscle.

Fat identification by means of regions of interest drawn on CT images (Figure 1a), allows attenuation differences of different tissues to be found. On MRI, fat identification relies on hyperintensity visible on *T*_1_ weighted images (Figure 1b) together with the fat supression technique (Figure 1c), which nulls the magnetization of fat at certain times during acquisition. We also performed a diffusio-weighted acquisition by means of “diffusion-weighted imaging with background suppression” pulse sequence acquired at four different crescent values of the “*b*” coefficient (maximum *b* = 800 s mm^–^²) to assess if the tissue had pathologic restriction of molecular water movement; no pathologic restriction was found (Figure 1d).

## Differential diagnosis

The very smooth and round contour of the lesion and the lack of contrast-enhancement suggested a benign lesion. A fat-containing mass can indicate the presence of a lipoma but the eventual presence of a calcified core can indicate the differential diagnosis of the lesion as a teratoma.

The clinical and histopathological differential diagnosis for ECMT includes several entities, such as benign mesenchymal proliferations with a myxoid component, focal fibrous hyperplasia, myofibroma, neurofibroma, granular cell tumour, schwannoma, leiomyoma and rhabdomyoma.^1^

The histopathological differential diagnosis has a wider spectrum and can also include many other lesions with a myxoid component, such as oral focal mucinosis, nerve sheath myxoma/neurothekeoma, soft tissue myxoma, glial choristoma and ossifying fibromyxoid tumour of the soft parts. The chondroid component may also allow consideration of other types including cartilaginous choristoma and extraskeletal myxoid chondrosarcoma.^1^

## Treatment

Transoral dissection by means of diode laser, extending from the left anterior tonsillar bundle to the soft palate, was followed by partial reconstruction *via* intramuscular suture. Oral feeding started on the third postoperative day.

## Outcome and follow-up

**Figure 2.**
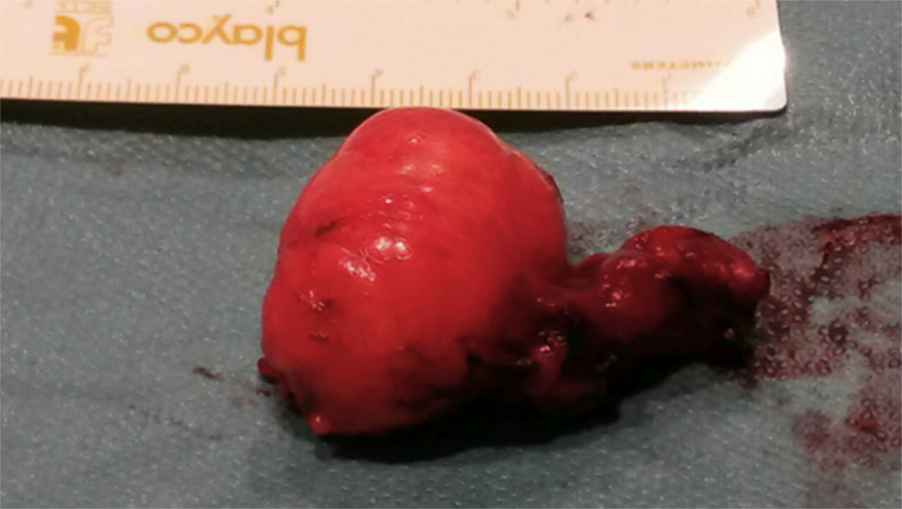
Surgical specimen showing the smooth and regular surface of the mass.

The surgical findings (Figure 2) were examined in the department of pathology, and on gross examination, a tender yellowish lesion nodule of soft elastic consistency with a central calcified area measuring 3.4 cm in the maximum diameter was seen (after decalcification before processing with a solution containing EDTA and hydrochloric acid).

Surgical specimens were fixed in 10% neutral buffered formalin and embedded in paraffin blocks. Histological sections were cut and stained with haematoxylin and eosin.

On light microscopy, the tumour showed polygonal and spindle-type cells, with uniform small nuclei and absence of mitoses on a chondromyxoid background with foci of osseous metaplasia.

At the microscopic level a well-circumscribed, unencapsulated cell proliferation of uniform round to fusiform shape in a chondromyxoid matrix was recognizable. The immunohistochemistry showed positivity for glial fibrillary acidic protein (GFAP) and frequent positivity for S-100 and cytokeratins.^2^

**Figure 3.**
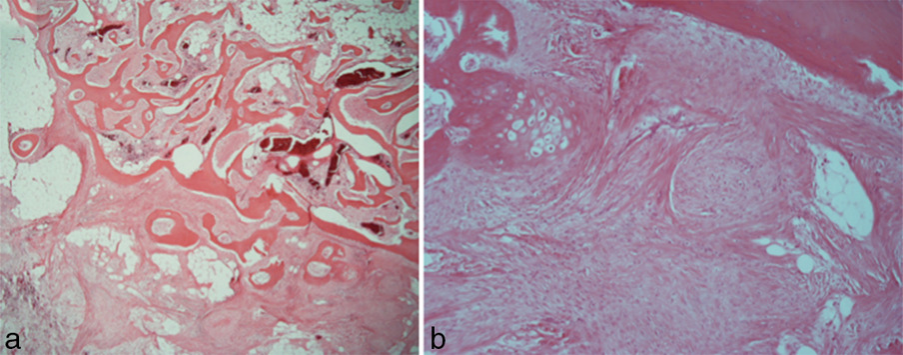
Haematoxylin and eosin staining. (a) Magnification at 20×; (b) magnification at 100×.

**Figure 4.**
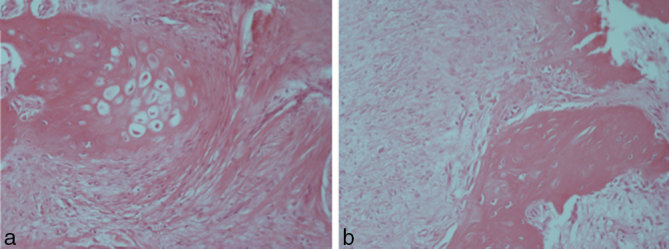
(a,b) Haematoxylin and eosin staining. Magnification at 200×.

Although the immunoprofile was negative for GFAP, S-100, cytokeratin AE1/AE3 and α-smooth muscle actin, most likely owing to the decalcification process, the final histological diagnosis was that of ECMT (Figures 3a,b and 4a,b). At the 6-month follow-up, the patient did not show symptoms or dysphagia.

## Discussion

ECMTs are rare, benign mesenchymal soft tissue neoplasms usually located in the oral cavity, particularly the tongue. The entity was first described in 1995 by Smith et al.^3^ They described 19 tumours, all localized to the anterior dorsal surface of the tongue. Despite the original description of ECMT, the clinical, histopathological and immunohistochemical features of this tumour have not been thoroughly defined as these are rare tumours, with only 48 ECMTs having been reported in the English literature. The histopathological similarity of several tumours such as myoepithelioma, oral focal mucinosis, soft tissue myxoma, ossifying fibromyxoid tumour, chondroid choristoma, nerve sheath myxoma, pleomorphic adenoma and mucocele results in only a small number of of ECMTs getting diagnosed.

Clinically, ECMT presents as a slow growing, asymptomatic swelling usually seen on the anterior dorsum of the tongue; however, two cases presenting on the posterior tongue have also been documented. In addition, a case of ECMT on the hard palate has been reported but owing to lack of appropriate supporting documentation, its diagnosis has been the subject of controversy. The size of the lesion usually varies from 0.3 to 2.0 cm and the age of patients ranges from 9 to 78 years, with a mean age of 39 years.^4^

No sex predilection is reported. The diagnosis of ECMT is based on the clinical as well as histopathological and immunohistochemical confirmation.^3^,^5^ The differential diagnosis of ECMT with other extraskeletal myxoid chondroma, oral focal mucinosis, pleomorphic adenoma, myoepithelioma, mucocele, glial/chondroid choristoma, and ossifying fibromyxoid tumour is not simple.^6^

Some features were reported in the literature, which allowsa differential diagnosis to be defined; for example, extraskeletal myxoid chondroma (ESMT) usually occurs in the hands or feet and is often seen related to tendon; myoepithelioma of minor salivary gland can show both chondroid and myxoid stroma; and ossifying fibromyxoid tumour shows the presence of bone in the periphery.^7^

ECMT probably arises directly from the neural cells of the tongue or primitive mesenchymal cells that undergo neural differentiation during oncogenesis.^3^

The expression of S-100 and GFAP supports a neurogenic origin.^8^ The tumour is negative for epithelial markers such as keratins and carcinoembryonic antigen; however, other authors have reported variable positivity for cytokeratin.^3^ No malignant change is reported and rare recurrence has been observed in the literature after complete excision.^2^

The treatment of choice is complete excision with tumour-free margins, and it is feasible because these lesions are small and relatively well-circumscribed.

The other few cases described in the literature did not include a complete integrated radiological work-upof the lesions reported. CT scan and MRI, by themselves, do not allow the diagnosis of ECMT to be hypothesized, although it may be suspected, but prove useful in diagnostic pathology, tissue characterization and therapeutic decision-making.gImaging helps mainly in defining the tumoral boundary, internal composition and determination of eventual infiltration into the surrounding structures.^9^

This case is of interest for several reasons: the rarity of the ECMT, the complete imaging work-up and the “tonsillar” site, with the majority of ECMT having been reported, until now, with "lingual" localization.

## Learning points

Oropharyngeal benign masses with central calcification and fat are usually considered benign at first, they can be misinterpreted as a teratoma (although unusual for that anatomical site), and the evidence for this rare type of tumour (ECMT) should be considered, in other infrequent cases of found at the tongue site. While imaging helps in defining and confirming the benign nature of a mass, histopathological features confirm its diagnosis.

## Patient’s perspective

The patient told us that she was very worried about cancer, but after CT scan and MRI, we could calm her because we were quite sure that the lesion was benign. The histopathological analysis allowed us to tell her that it was not malignant, nor would it recur.

## Consent

Written informed consent was obtained from the patient for publication of this case report, including accompanying images.
